# Visual Imagery and Perception Share Neural Representations in the Alpha Frequency Band

**DOI:** 10.1016/j.cub.2020.04.074

**Published:** 2020-07-06

**Authors:** Siying Xie, Daniel Kaiser, Radoslaw M. Cichy

**Affiliations:** 1 Department of Education and Psychology, Freie Universität Berlin, Habelschwerdter Allee 45, Berlin 14195, Germany; 2 Department of Psychology, University of York, Heslington, York YO10 5DD, UK; 3 Berlin School of Mind and Brain, Humboldt-Universität zu Berlin, Unter den Linden 6, Berlin 10099, Germany; 4 Bernstein Centre for Computational Neuroscience Berlin, Humboldt-Universität zu Berlin, Unter den Linden 6, Berlin 10099, Germany

**Keywords:** mental imagery, object perception, feedback, oscillations, deep neural networks

## Abstract

To behave adaptively with sufficient flexibility, biological organisms must cognize beyond immediate reaction to a physically present stimulus. For this, humans use visual mental imagery [1, 2], the ability to conjure up a vivid internal experience from memory that stands in for the percept of the stimulus. Visually imagined contents subjectively mimic perceived contents, suggesting that imagery and perception share common neural mechanisms. Using multivariate pattern analysis on human electroencephalography (EEG) data, we compared the oscillatory time courses of mental imagery and perception of objects. We found that representations shared between imagery and perception emerged specifically in the alpha frequency band. These representations were present in posterior, but not anterior, electrodes, suggesting an origin in parieto-occipital cortex. Comparison of the shared representations to computational models using representational similarity analysis revealed a relationship to later layers of deep neural networks trained on object representations, but not auditory or semantic models, suggesting representations of complex visual features as the basis of commonality. Together, our results identify and characterize alpha oscillations as a cortical signature of representations shared between visual mental imagery and perception.

## Highlights

• Perception and imagery share neural representations in the alpha frequency band
• Shared representations stem from parieto-occipital sources
• Modeling suggests contents of shared representations are complex visual features

## Teaser

Visual imagination is a key cognitive function with the feel of weak visual perception. Xie et al. show that this phenomenal likeness is mirrored by neural likeness in shared representations of complex visual features in the alpha frequency band activity arising from parieto-occipital cortex.

## Results and Discussion

**Figure 1:**
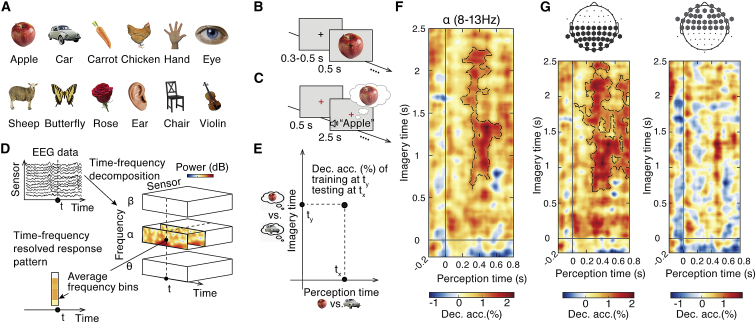
Methods and Results of Multivariate Classification Analyses (A) Stimuli were a diverse set of twelve object images and twelve spoken words denoting these objects. (B) In the perception task, participants viewed the object images in random order. (C) In the mental imagery task, participants were cued to imagine an object by hearing the spoken word denoting the object. (D) EEG data recorded from 64 electrodes during both tasks were epoched into trials and subjected to time-frequency decomposition using Morlet wavelets. This was done separately for each single trial and each electrode, yielding a trial-wise representation of induced oscillatory power. We aggregated these time-frequency data into three frequency bands (theta: 5–7 Hz; alpha: 8–13 Hz; beta: 14–31 Hz). Averaging across all frequencies within each band yielded a time- and frequency-resolved response vector (across EEG sensors) for each trial. These response vectors were entered into multivariate pattern analyses. (E) Multivariate pattern classification was performed separately for each frequency band. As perception and imagery need not emerge with similar temporal dynamics, we performed a time-generalization analysis in which we considered timing in the perception and imagery tasks independently. For every time point combination during perception (0–800 ms with respect to image onset) and imagery (0–2,500 ms with respect to word onset) separately, we conducted a pairwise cross-classification analysis where we trained support vector machine (SVM) classifiers to discriminate between response patterns for two different objects (here: car versus apple) when they were imagined and tested these classifiers on response patterns for the same two objects when they were perceived (and vice versa). We averaged classification accuracies for all pairwise classification analyses between objects, yielding a single time-generalization matrix for each frequency band. These matrices depict the temporal dynamics of representations shared between imagery and perception. (F) We found significant cross-classification in the alpha frequency band, ranging from 200 to 660 ms in perception and from 600 to 2,280 ms in imagery. Peak decoding latency was at 480 ms (95% confidence intervals: 479–485 ms) in perception and 1,340 ms (95% confidence intervals: 1,324–1,346 ms) in imagery. (G) To spatially localize these shared representations, we performed separate time-generalization analyses for anterior and posterior electrodes in our EEG setup. This analysis revealed significant cross-classification in the alpha band for posterior electrodes (from 20 to 800 ms during perception and from 660 to 2,500 ms during imagery), but not in the anterior electrodes. This suggests that parieto-occipital alpha sources mediate the shared representations between perception and imagery. Black outlines indicate time point combinations with above-chance classification (N = 38; non-parametric sign permutation tests; cluster-definition threshold p < 0.05; cluster threshold p < 0.05; Bonferroni corrected by 3 for the number of frequency bands tested). Dec. acc., decoding accuracy. See also Figure S1.

Imagining and perceiving visual contents recruits similar brain circuits [1, 3] with related neural dynamics [4, 5]. However, the temporal dynamics of neural mechanisms mediating this similarity as well as their nature remain less well understood. To characterize these neural mechanisms and their temporal fingerprint, we recorded brain responses with high temporal resolution using electroencephalography (EEG) and analyzed them using multivariate pattern analysis [6, 7, 8] and model comparison through representational similarity analysis [9, 10, 11]. We analyzed brain responses to twelve different visual objects (Figure 1A) that participants (N = 38) either viewed as images (perception task; Figure 1B) or visually imagined after a spoken word cue (imagery task; Figure 1C).

How do neural representations shared between imagery and perception emerge? Unlike perception, imagery lacks feedforward information flow from the stimulus, suggesting that neural representations shared between imagery and perception emerge through feedback information flow. Feedforward and feedback information in the visual brain are carried by different neural oscillation channels: theta and gamma oscillations carry feedforward information, and alpha and beta oscillations carry feedback information [12, 13]. We thus expected representations shared between imagery and perception to emerge in the alpha or beta frequency range.

To determine the temporal dynamics of neural representations in particular frequency bands, we used multivariate pattern analysis (MVPA) on time-frequency resolved EEG data (Figures 1D and 1E). The general rationale is that if imagined and perceived objects evoke similar neural activity, a classifier trained to discriminate objects based on neural activity during imagery will successfully classify these objects from neural activity during perception and vice versa. Note that there is no reason to assume that neural processing for imagery takes the exact same time course as for perception—instead, neural processing could be delayed [4], slowed [14], or even reversed [5]. We therefore used a time-generalization variant of MVPA, evaluating classifier performance on all possible time point combinations for perception (0–800 ms after image onset) and imagery (0–2,500 ms after sound onset). Separately for each frequency of interest (Figure 1D), this resulted in a two-dimensional classification accuracy matrix identifying time-point combinations, during which neural representations are similar between imagery and perception (Figure 1E).

### Imagery and Perception Share Neural Dynamics in the Alpha Frequency Band

The key result is that imagery and perception share neural dynamics in the alpha frequency band (Figure 1F; for timing, see figure caption), but not in the theta or beta frequency band (Figures S1A–S1C), even though we found strong object classification in all frequency bands when analyzing perception and imagery data separately (Figures S1H–S1J). This result was robust to several analysis choices: it was independent of changes in particular data aggregation choices for the multivariate pattern analysis (Figures S1D–S1F and S1K–S1M); it held for both possible directions of cross-classification analysis (from imagery to perception and vice versa; Figures S1O and S1P); it was achieved when imagery data were temporally aligned not to sound onset but to sound offset (Figures S1Q–S1S); and it held also when participant-specific alpha frequencies were used (Figures S1T–S1V). Moreover, we found no shared neural dynamics in broadband (evoked) responses (Figure S1G), although they also contained robust object information when imagery and perception were analyzed separately (Figure S1N).

Our finding adds to previous research on shared representations between imagery and perception [1, 2, 4, 15, 16, 17, 18, 19] by specifically identifying oscillations in the alpha frequency band as a neural signature of representations shared between imagery and perception. In particular, our findings go beyond previous work suggesting that alpha oscillations play a role in mental imagery [20, 21] by specifying a dedicated role of alpha oscillations in the encoding of particular visual contents.

Our findings characterize the temporal dynamics of the shared representations. For one, we find that shared representations emerge relatively later in imagery than in perception. This reinforces the notion that imagery and perception differ in their information-processing dynamics [4, 14]. However, in our design, participants had longer time (2,500 ms) to imagine the stimulus than they had to perceive it (500 ms), which might have influenced the dynamics. Systematic investigations using speeded versus non-speeded imagery tasks are necessary to further investigate this observation. A further observation is that shared representations arise relatively late in the time course of visual processing. They thus unlikely reflect early sensory processing in the first feedforward pass that is more rapid [6, 22, 23, 24]. Finally, the cross-classification analysis generalizes over long periods of time, suggesting persistent rather than transient neural dynamics. Persistent processing has previously been observed during late visual processing and has been linked to high-level ventral visual cortex [6]. Alternatively, the timing observed here is also consistent with neuronal latencies of visual processing in memory-related circuits of the medial temporal lobe [25], as well as the timing of reinstatement of encoded memory signals during retrieval [26, 27, 28, 29, 30] that also show persistence [31, 32, 33].

A large body of research has indicated that alpha oscillations in the brain are not a unified but diverse phenomenon, implicated in many cognitive functions and reflecting different mechanisms and brain networks [34, 35, 36, 37, 38, 39, 40, 41]. One key distinction is between parieto-occipital alpha mechanisms implicated more in perceptual functions and frontal alpha implicated more in cognitive functions, such as executive control [42]. To investigate whether the shared representations between imagery and perception are related to one or the other, we repeated the analysis restricted to either anterior or posterior EEG electrodes (Figure 1G). The cross-classification analysis in the alpha band yielded significant cross-classification for the posterior, but not the anterior, EEG electrodes. A supplementary analysis of classifier weight distributions across the scalp provided consistent evidence, highlighting posterior EEG electrodes as most strongly relevant for classification in both imagery and perception (Figures S1X and S1Y). This lends further support to the idea that shared representations between imagery and perception are more strongly related to parieto-occipital alpha oscillations implicated in perceptual functions.

Together, our finding that imagery and perception share representations in the alpha frequency band from parieto-occipital sources has two implications. First, they elucidate the neural mechanisms of conscious phenomenal experience. Visually imagined contents are subjectively felt to be similar to perceived contents, and our results suggest that alpha oscillations play a role in mediating this subjective similarity. Second, our findings advance our general understanding of alpha oscillations. They are hard to reconcile with the view that alpha oscillations reflect cortical idling [43] or inhibition of irrelevant information [35, 44, 45], as we find them to encode task-relevant contents in memory and perception. Instead, our results are more aligned with the idea that alpha oscillations can have an active role in information processing. Recent evidence for this view stems from research in feedback communication [12, 13], working memory [26], and memory [39, 46]. However, note that our study established the encoding of contents in the alpha frequency band rather than a relationship between the fidelity of encoding and net alpha power (that did not predict shared information between imagery and perception in our study; Figure S1W). Further research is needed to unravel the relation between net alpha power changes and the information alpha band oscillations contain.

A remaining open question is whether shared representations indeed reflect feedforward- or feedback-related processing in perception. This question cannot be addressed with our data alone. However, the fact that we find shared representations in the alpha band, which is generally associated with top-down processing, speaks for a role of feedback. Future studies comparing imagery to perception with reduced feedback processing, e.g., through masking [47, 48, 49], with imagery could shed light on this issue.

One limitation of our study is that our analysis depends on strong averaging of individual trials (see STAR Methods). We chose to apply averaging because of the potentially low signal-to-noise ratio (SNR) of imagery signals and because single-trial imagery responses may be strongly dispersed in time and thus only highly averaged data can reveal them. This averaging does not impede the main conclusions of the current paper, but it should be noted that we did not decode the content of participant’s mental imagery on a single-trial basis. To achieve accurate single-trial classification, future research might increase detection power by employing a design that allows time locking the analysis to the end rather than the beginning of imagery [5] or by using classification methods that take temporal variability in single trials into account [50].

### The Format of Shared Representations in the Alpha Frequency Band

**Figure 2:**
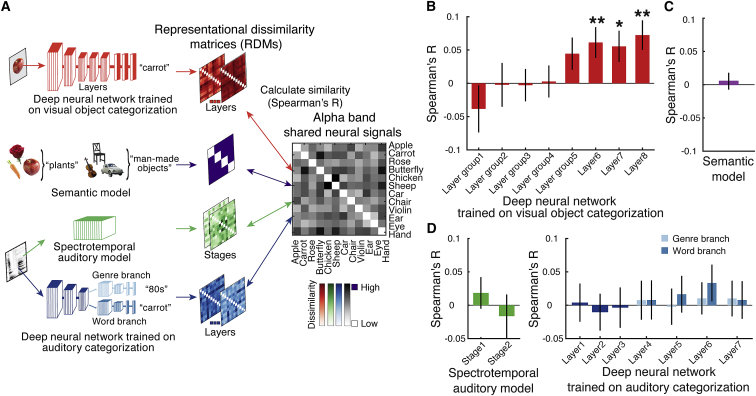
Methods and Results of Relating Shared Representations to Computational Models (A) We characterized the format of the representations shared between imagery and perception in the alpha frequency band by relating EEG signals to computational models using representational similarity analysis [10, 11]. For each participant, we first constructed a 12 × 12 neural representational dissimilarity matrix (RDM) that contained the pairwise cross-classification accuracy between imagery and perception for each possible object pair (data, models, and results are color-coded similarly; EEG data here in gray). This summarizes the representational geometry of the shared representations between imagery and perception in the alpha band. We then related (Spearman’s R) neural RDMs to model RDMs that captured hypotheses about the format of the shared representations: (1) a deep neural network (DNN) trained on visual object classification (VGG-19 [56]; color-coded red) to assess visual processing; (2) a category model that captures superordinate-level category membership of the objects in 4 categories (animals, body part, plants, and man-made objects; color-coded purple) to assess semantic processing; and (3) a spectrotemporal auditory model [57] (color-coded green) and a DNN with two branches trained on musical genre and auditory word classification, respectively [58] (color-coded blue) to assess auditory processing. Visualizations of all model RDMs can be found in Figures S2A–S2C. (B–D) We found a significant relationship between neural and model RDMs only for the late layers of the DNN trained on visual object classification (B), but not for the semantic model (C) or the auditory models (D). Error bars represent standard errors of the mean. Asterisks indicate significant correlations between model RDMs and neural RDMs (N = 38; non-parametric sign-permutation tests; ^∗^p < 0.05; ^∗∗^p < 0.01; false discovery rate [FDR] corrected for multiple comparisons across RDMs per model).

Although the cross-classification analysis established that imagery and perception share representations of particular contents, it cannot by itself tell the format of these representations, i.e., which features of the objects are shared in the representations [11, 14, 51, 52, 53, 54]. One possibility is that the signal indexes shared visual representations of low- or high-level features [1, 3, 15, 16, 55]. Another one is that it reflects category membership that is abstract and semantic in nature. A third possibility is that it reflects verbal representations, for instance, because participants silently vocalize the word cue they hear in the imagery task during the perception task. To arbitrate between these possibilities, we operationalized them in computational models that we compared to alpha-frequency signals in the EEG using representational similarity analysis [10, 11] (Figure 2A; models and respective results color-coded; EEG data in gray). We used the following models (Figure 2A): (1) as a visual model, we used a deep neural network (DNN) model trained on visual object classification (VGG-19, color-coded red) [56]; (2) as a semantic category model, we used an explicit category model that captures the objects’ membership in four superordinate-level categories (animals, body parts, plants, and man-made objects; color-coded purple); and (3) as auditory models, we used both a commonly used spectrotemporal model [57] (color-coded green) and a DNN model trained on auditory word and musical genre classification [58] (color-coded blue). In brief, we found that the higher layers of the DNN trained on visual object categorization were related to representations shared between imagery and perception in the alpha frequency band (Figure 2B), but not the semantic model (Figure 2C) or the auditory models (Figure 2D). This difference was not trivially related to one model generally fitting its associated domain better than the other: as expected, we found robust and significant fits between the visual DNN with the perception data (Figure S2D) and between the auditory models and the imagery data (Figure S2E). Together, this suggests that the shared representations between imagery and perception in the alpha frequency band are of complex visual features as found in the high layers of DNNs trained on object recognition.

High processing layers of visual DNNs contain high-dimensional representations of whole objects and object parts [59, 60, 61, 62, 63, 64] and predict brain activity particularly well in high-level visual cortex [65, 66, 67, 68, 69, 70] (but see also [71]). Our results therefore suggest that shared representations between imagery and perception in the alpha band are representations of complex visual features as encoded in high-level visual cortex. This further refines the conclusion that the shared representations between imagery and perception originate from posterior brain regions, potentially reflecting activations in high-level ventral visual cortex. By contrast, we did not observe a relationship to early DNN layers, and thus our results do not allow conclusions about the role of low-level visual cortex. This might be so as low-level visual cortex might have been activated by both imagery and perception, but in different ways, and thus not captured by our cross-decoding approach. Further, activation of early visual cortex in imagery has been observed to depend on factors such as task [72] and vividness [73], for which our design was not optimized. Future research is needed to reveal the role of oscillatory activity in imagery, taking these factors into explicit account.

Our results suggest that the observed cross-classification cannot be easily explained by effects of spatial attention only. Spatial location of stimuli perceived or kept in memory can be decoded from the alpha frequency band [26, 41, 74], suggesting that cross-classification of objects between imagery and perception might reflect spatial attention to particular visual features in perception, and their likely location in the feedback flow during imagery. However, no attentional mechanisms are implemented in the object-classification DNN used here. Future studies that manipulate both content and location of imagery [55] could further disentangle the role of those factors.

The implications of our study are limited in principle by the nature and size of the stimulus material used to probe brain activity. Visual-imagery-related brain signals are low in SNR overall, and imagery studies thus commonly focused on two-category designs, such as places versus faces [15] or animate versus inanimate objects [5].

Even most encoding model studies that harvested the higher SNR of veridical perceptual signals for model training, allowing them to use a large number of conditions (even >1,000), tested the model on a much smaller number of conditions (5 or 6) in imagery [75, 76]. Here, we choose a rather diverse set of twelve everyday objects that differed widely in their shape, color, and orientation. Their pairwise comparison resulted in 66 values that offer a rich characterization of the brain dynamics. In fact, it was our relatively diverse stimulus set that allowed us to arbitrate different contents of shared representations. In particular, our finding that representations shared between imagery and perception in the alpha frequency band were representationally similar to a DNN trained on object categorization points toward a promising venue: magneto- and electroencephalography (M/EEG) studies using an encoding approach based on DNNs akin to a recent fMRI study [77] might be able to distinguish larger numbers of stimuli.

### A Neural Signature of Representations Shared between Imagery and Perception

In sum, our results identify and characterize the oscillatory signature of representations shared between visual mental imagery and perception. We find that shared representations of objects are present in the alpha frequency band, they originate from posterior locations in the brain, and they are similar in format to representations in higher layers of visual deep neural networks. By identifying alpha oscillations as a neural mechanism mediating the perceived subjective similarity between visual imagery and perception, our findings elucidate the neural mechanisms of conscious phenomenal experience. They also further our understanding of brain oscillations, suggesting that activity in the alpha frequency band plays an active role in cortical communication by mediating visual contents.

## STAR★Methods

### Key Resources Table

**Table:** 

REAGENT or RESOURCE	SOURCE	IDENTIFIER
**Deposited Data**		

Raw and analyzed data	This paper	https://osf.io/ykp9w/

**Software and Algorithms**		

MATLAB	Mathworks Inc.	https://www.mathworks.com/products/matlab.html; RRID: SCR_001622
Psychtoolbox	[79]	http://psychtoolbox.org/; RRID: SCR_002881
Brainstorm	[80]	https://neuroimage.usc.edu/brainstorm/; RRID: SCR_001761
LIBSVM Toolbox	[81]	https://www.csie.ntu.edu.tw/∼cjlin/libsvm/; RRID: SCR_010243
MatConvNet MATLAB Toolbox	[82]	https://github.com/vlfeat/matconvnet
NSL MATLAB Toolbox	[52]	http://nsl.isr.umd.edu/downloads.html
Deep neural network trained on auditory categorization	[53]	https://github.com/mcdermottLab/kelletal2018

### Resource Availability

#### Lead contact

Further information and requests for resources, data and code should be directed to and will be fulfilled by the Lead Contact, Radoslaw Martin Cichy (rmcichy@zedat.fu-berlin.de).

#### Materials availability

This study did not generate new unique reagents.

#### Data and code availability

The dataset generated during this study is available at OSF, https://doi.org/10.17605/OSF.IO/YKP9W.

### Experimental Model and Subject Details

38 healthy participants with normal or corrected-to-normal visual acuity (age: mean ± SD = 24.1 ± 4.99 years, 30 female) participated in the study. All procedures were approved by the ethical committee of the Freie Universität Berlin and conducted in accordance with the Declaration of Helsinki. Participants gave written informed consent and received either money or course credits for compensation.

### Method Details

#### Stimuli

The stimulus set consisted of a set of object images and audio recordings of a human voice uttering their corresponding German names. The image set comprised 12 silhouette color photographs of everyday objects on a gray background (Figure 1A). In addition to these 12 objects, an image of a paper clip was used as a target stimulus in catch trials of the perception task (see below). The audio recordings were 12 spoken German words taken from a German standard dictionary website (*Duden*, https://www.duden.de), with each word corresponding to one of the object images. Each recording was digitized at a 44.1 kHz sampling rate and normalized by their root mean squared amplitude. The average duration of the sound recordings was 554.3ms (SD: ± 17.8ms).

#### Experimental design

The experiment consisted of two identical recording sessions, performed on two different days. Within each session, participants first completed the perception task (Figure 1B) and then the mental imagery task (Figure 1C). Additionally, they completed a third, auditory task, which was related to a different research question, and is not reported in the current manuscript. Experimental stimuli were delivered using Psychtoolbox [79].

In the perception task, participants viewed the object images. On each trial, one of the object images (∼2.9° visual angle) was presented for 500 ms at the center of the screen, overlaid with a black fixation cross. Participants were instructed to press a button and blink their eyes when the image of the paper clip appeared (on average every 5^th^ trial). Trials were separated by an inter-trial interval (ITI) of 300 ms, 400 ms, or 500 ms, during which only the fixation cross was presented. Participants were instructed to maintain central fixation throughout the experiment. Following catch trials, the ITI was lengthened by 1000 ms to avoid contaminating the subsequent trial with motor artifacts. In each recording session, participants completed 600 trials of the perception task, split into two blocks separated by a self-paced break.

In the mental imagery task, participants were presented with the audio recordings of the words and were asked to actively imagine the object corresponding to the word they had heard. Each trial started with a red fixation cross, 500 ms after which the audio recording of an object name was played. Participants were instructed to visually imagine the corresponding object image as soon as they heard the object name for 2,500 ms. After the imagery period, participants indicated whether the vividness of their mental image was high or low by selecting one of two letters (H versus L) on a 1,500 ms response screen. The positions of the response options were counterbalanced across trials. Participants indicated high vividness of their imagery in the majority of trials (83.4%, SD: ± 0.09), indicating that, subjectively, participants formed precise mental images of the objects. Trials were separated by an ITI of 300 ms, 400 ms or 500 ms, where a black fixation cross was presented. In each recording session, participants completed 480 trials of the imagery task, split into four blocks interrupted by self-paced breaks.

To familiarize participants with the mental imagery task and to make sure they could vividly imagine the objects, we trained them in imagining our object images prior to the mental imagery task. During this training procedure, participants practiced imagining the 12 objects after hearing audio recordings of their names. On each trial of the training procedure, participants first viewed one of the object images for as long as they wished, in order to familiarize themselves with the image. After they were confident that they could imagine the object, they proceeded to the 2,500 ms imagery period, where they first heard the audio recording and then imagined the object. After this imagery period, participants again viewed the object image for as long as they wished, in order to self-evaluate the correctness of their imagery. After each object was trained once (i.e., after 12 trials), participants entered a two-alternative forced-choice test procedure, where on each trial (one for every object, i.e., 12 trials) an object image from the stimulus set was presented alongside with a very similar foil image not from the stimulus set. Foil images were drawn randomly from a set of 3 alternatives. If participants achieved 80% correct in this test, they proceeded to the main experiment. If participants failed to achieve 80% correct, the training procedure and the subsequent test were repeated until the participant reached 80% correct.

#### EEG acquisition and preprocessing

EEG data was recorded using an EASYCAP 64-channel system and Brainvison actiCHamp amplifier. The 64 electrodes were arranged in accordance with the standard 10-10 system. Acquisition was continuous with a sampling rate of 1000 Hz and the EEG data was filtered online between 0.3 and 100 Hz. All electrodes were referenced online to the Fz electrode. Offline preprocessing was carried out using Brainstorm [80]. Eyeblinks and eye movements were detected and removed with an independent component analysis on frontal electrodes Fp1, Fp2, AF7 and AF8 in the 64-channel EASYCAP system as implemented in the ‘SSP: Eye blinks’ (Signal-space projection) algorithm in Brainstorm. We visually inspected the components and removed those resembling the spatiotemporal properties of eyeblinks and eye movements. The number of components removed was between one and four for each participant, and a clear eye-blink component was always found and removed. To avoid edge artifacts in the subsequent time-frequency decomposition, the continuous EEG raw data was extracted in epochs between 600 ms pre-stimulus and 1100 ms post-stimulus in the visual perception task and between 600 ms pre-stimulus and 3100 ms post-stimulus in the mental imagery task. For the main analysis data were time-locked to the onset of the visual image in the perception task and to the onset of the auditory word in the imagery task; time-locking the imagery data to the offset of each word yielded qualitatively similar results in the key analyses described below (Figures S1Q–S1S). The epoched data was baseline-corrected by subtracting the mean of the pre-stimulus interval, separately for each channel and trial.

#### Time-frequency decomposition

EEG data recorded for the visual perception task and for the mental imagery task were analyzed separately. To recover induced oscillatory responses, the data was convolved with complex *Morlet* wavelets (constant length of 600 ms, logarithmically spaced in 20 frequency bins between 5 Hz and 31 Hz), separately for each trial and each sensor. By taking the square root of resulting time-frequency coefficients, we obtained the absolute power values for each time point and each frequency between 5Hz and 31 Hz. These power values were normalized to reflect relative changes (expressed in dB) with respect to the pre-stimulus baseline (−500 ms to −300 ms relative to stimulus onset). To increase the signal-to-noise ratio of all further analyses, we downsampled the time-frequency representations to a temporal resolution of 50 Hz (by averaging data in 20 ms-bins) and aggregated the 20 frequency bins into three discrete frequency bands (which we analyzed separately): theta (5-7 Hz, 5 bins), alpha (8-13 Hz, 6 bins) and beta (14-31 Hz, 9 bins).

#### Classification of oscillatory responses

To uncover shared representations between perception and imagery, we trained classifiers to discriminate pairs of objects from EEG data recorded during one task (i.e., perceiving an apple versus perceiving a car) and tested them on EEG data recorded for the same two objects in the other task (i.e., imagining an apple versus imagining a car). Above-chance classification performance in this cross-task procedure indicates that similar representations are evoked by imagining and perceiving objects. Classification was performed in a time- and frequency band-resolved fashion, that is separately for each frequency band and each time point. This allowed us to quantify (1) which frequency bands mediate these shared representations, and (2) with which temporal dynamics these representations emerge.

The detailed steps of the procedure are as follows. First, the data for each trial, each frequency band, and each time point was unfolded into a single pattern vector. For this, the data was averaged across frequencies contained in the frequency band (e.g., for the 6 frequency bins between 8 and 13 Hz for the alpha band), yielding a 63-element pattern vector (i.e., one value for each electrode). Note that results did not depend on the particulars of how data was aggregated in the frequency domain: A control analysis in which we, instead of averaging across the frequency bins in each band, concatenated the data across all frequency bins (e.g., 6 frequency bins × 63 electrode pattern vectors for the alpha band) yielded qualitatively equivalent results (Figures S1G–S1L).

Second, we created four pseudo-trials for every condition by averaging pattern vectors across trials where the same object was shown in the same task: for example, this resulted in four pseudo-trials for the apple in the imagery task, each constituting the average of 25% of the available trials (assigned randomly).

Third, we trained and tested linear support vector machines (C-SVC with a linear kernel and a cost parameter of c = 1, as implemented in the *libsvm* package [81]) using those pseudo-trials. This classification was performed across tasks: For each pairwise combination of objects, we trained classifiers to discriminate the objects using the four pseudo-trials in one task (e.g., the perception task). Then we tested these classifiers on the same two objects using data from the four pseudo-trials in the other task (e.g., the imagery task). Classification was repeated across both train-test directions (i.e., train on perception and test on imagery data, and train on imagery and test on perception data) and across all pairwise object combinations, and classifier performance (i.e., classification accuracy) was averaged across these repetitions. Averaging was performed along the “perception” and “imagery” axes of both analysis variants, so that a successful generalization from perception at 200ms to imagery at 800ms ended up at the very same point in the time generalization matrix, independently of the train-test direction. Results were consistent across both train-test directions (Figures S1O and S1P). Finally, the whole classification analysis was repeated 100 times, with new random assignments of trials into pseudo-trials, and results were averaged across these 100 repeats.

Importantly, as the temporal dynamics of cortical responses to perceived and imagined objects are not expected to be identical (e.g., responses during imagery could be delayed, slowed or reversed), we performed classification analyses in a time-generalization fashion [8]. That is, we did not only train and test classifiers on the same time points with respect to stimulus presentation, but we trained and tested classifiers on each combination of time points from the perception task (i.e., from 0 to 800 ms with respect to image onset) and the imagery task (i.e., from 0 to 2,500 ms with respect to sound onset). The analysis thus yielded time generalization matrices that indicate how well classifiers trained at one particular time point during perception perform at each time point during imagery (and vice versa). The resulting time-generalization matrices thereby yielded a full temporal characterization of shared representations between perception and imagery, separately for each of the three frequency bands (Figures S1A–S1C and S1D–S1F for alternative data aggregation method).

In addition to the cross-task classification analysis, we also performed a within-task classification analysis where we classified objects from EEG data recorded within one task, i.e., solely for the perception task or solely for the imagery task, again separately for each frequency band. This analysis was carried out in the same way as the cross-classification analysis (see above) with a leave-one-pseudo-trial-out cross-validation scheme: We trained classifiers to discriminate two objects using data from three of the four pseudo-trials and then tested these classifiers using data from the remaining, the fourth pseudo-trial. Classification was repeated 100 times, with new random assignments of trials into pseudo-trials, and results were averaged across these 100 repeats. For the within-task classification analyses, we yoked training and testing times, leading to a time course of classification accuracies for each frequency band and task (Figures S1H–S1M).

In the main analyses we chose a pre-defined, canonical range of frequencies to define the alpha band (8-13 Hz). However, peak alpha frequencies may vary between participants [78], suggesting that participant-specific alpha band should be defined separately for each participant. To determine the role of varying individual alpha frequencies on our analysis, we performed the cross-classification analysis based on each participant’s individual peak alpha frequencies and respective alpha band definitions. We defined participant-specific peak frequencies and respective bands using the following procedure. We first computed object classification on data from the perception task only, considering data at each frequency between 8 and 13 Hz with 1 Hz resolution and its two immediate neighbor frequencies (i.e., for 9 Hz including 8 and 10 Hz). For each participant, the peak alpha frequency was the frequency where within-task object classification accuracy was highest. The respective participant-specific frequency band was defined as the peak frequency and its two immediate neighbor frequencies (i.e., for peak frequency at 8 Hz the band is 7–9 Hz). We then repeated the cross-classification analysis using these participant-specific alpha frequencies. This yielded qualitatively similar results to the analysis based on the canonical alpha frequency band (see Figures S1T–S1V).

To determine whether cross-classification is enabled by large scale net increases or decreases in alpha power, we performed an additional analysis, in which we binned trials in the perception task according to whether they exhibited an increase or a decrease in alpha power, relative to baseline. We then re-performed the cross-classification analysis using only data from the perception task that either showed an alpha power enhancement (45% of trials) or an alpha power suppression (55% of trials). We equalized the number of trials by subsampling the alpha suppression trials to avoid bias. This analysis revealed no significant differences between alpha-enhanced or alpha-suppressed trials (Figure S1W).

#### Localization of shared representations

To investigate whether alpha-band representations shared between perception and imagery are related to parieto-occipital alpha or frontal alpha mechanisms, we conducted separate cross-classification analyses using either the anterior or the posterior halves of electrodes in our EEG montage. The anterior half consisted of the 35 electrodes located on the frontal, temporal and central parts of scalp, covering the Fp, AF, F, FT, T, and C channels in the EASYCAP 64-channel system. The posterior half consisted of 37 electrodes covering occipital and parietal cortex, covering the C, T, CP, P, TP, PO and O channels. The central and temporal channels were included in both halves. For both analyses, classification procedures were the same as described for the analysis including all electrodes.

As an additional measure of spatial localization, we examined the distribution of classifier weights obtained from training classifiers on data from all sensors. During classification analysis, each feature (i.e., here each EEG electrode) is assigned a weight corresponding to the degree to which its output is used by the classifier to maximize class separation. Therefore, classification weights index the degree to which different electrodes contain class-specific information. To directly compare the weights of electrodes across time, we transformed weights into activation patterns by multiplying them with the covariance in the training dataset [83]. For display purposes, we projected the reconstructed activation patterns onto a scalp topography (Figures S1X and S1Y). This analysis of classifier weights was done twice: once for classifiers trained on data from the perception task, and once for classifiers trained on data from the imagery task. We thereby obtained two sets of classifier weights across the scalp and across time, which allowed us to localize features relevant for detecting shared representations in sensor space.

#### The format of shared representations

To characterize the nature of the representations shared between imagery and perception we used representational similarity analysis [10, 11] in combination with computational models. The basic idea is that representations shared between imagery and perception are related to representations in computational models if they treat the same conditions as similar or dissimilar. To determine this, in a first step condition-specific multivariate patterns in the neural (here: EEG sensor patterns) and the model (e.g., model unit activation patterns) coding spaces are compared for dissimilarity independently. Dissimilarity values are aggregated in so-called representational dissimilarity matrices (RDMs) indexed in rows and columns by conditions compared (here: 12 × 12 RDMs indexed by the 12 objects). In a second step the neural RDMs and model RDMs are then related to each other by determining their similarity. We described the detailed procedures to construct neural and model RDMs as well as their comparison below.

The procedure to construct neural RDMs was as follows. Classification accuracy can be interpreted as a dissimilarity measure on the assumption that the more dissimilar activation patterns are for two conditions, the easier they are to classify [6, 84]. Classification accuracy at each time point combination in the cluster indexing shared representations between imagery and perception (Figure 1F) is the average of a 12 × 12 matrix of cross-classification accuracies for all pairwise object combinations. Here, instead of averaging across its entries, we extracted the full 12 × 12 RDM for each time point in the cluster and averaged the RDMs across all time-point combinations, yielding a single RDM for each participant. Thus, each participant’s RDM indicates the dissimilarity for object representations shared between imagery and perception.

To characterize the nature of these shared representations we extracted model RDMs from a set of computational models. These models mirrored the objects’ (i) visual dissimilarity, (ii) their semantic category dissimilarity, and (iii) their auditory dissimilarity (i.e., the dissimilarity of the word sounds used to cue imagery). The construction of model RDM was as follows.

As the visual model, we used the 19-layered deep convolutional neural network (DNN) VGG19 [56] pretrained to categorize objects of the ImageNet dataset [85]. Using the MatConvNet toolbox [82], we ran the 12 object images used in this study through the DNN and then constructed layer-specific model RDMs by quantifying the dissimilarity (1-Pearson’s R) of response patterns observed along each of the 19 layers of the DNN. We constructed 8 aggregated RDMs from these results. The first five RDMs were constructed from convolutional layers, averaging RDMs of convolutional layers positioned between max pooling layers, starting with the input layer (RDM1: convolutional layers 1,2; RDM2: convolutional layers 3,4; RDM3: convolutional layers 5-8; RDM4: convolutional layers 9-12; RDM 5: convolutional layers 13-16). The last three RDMs were constructed from activations in the three final fully connected layers each (RDM6-8).

For the semantic category model, we modeled category membership in a binary way. For this model, we split our 12 objects into four sets of superordinate-level category membership: animals (butterfly, chicken, sheep), body parts (ear, eye, hand), plants (apple, carrot, rose), and man-made objects (car, chair, violin). We then constructed a model RDM in which objects of the same category were coded as similar (−1) and objects from different categories were coded as dissimilar (+1).

We considered two auditory models: a canonical spectrotemporal model inspired by psychoacoustical and neurophysiological findings in early and central stages of the auditory system [57], and a DNN with two branches trained on musical genre and auditory word classification respectively [58]. We ran all word sounds used in this study through the spectrotemporal and the auditory DNN. We constructed auditory model RDMs by quantifying the dissimilarity of response patterns (1-Pearson’s R) observed in the 2 stages (i.e., auditory spectrograms and estimated cortical spectrotemporal features) of the spectrotemporal model and the 11 layers along the auditory DNN (i.e., 3 early shared convolutional layers and 4 layers (the first two convolutional, the latter two fully connected) along the two branches trained on genre and word classification respectively).

To quantify how well the different models were related to the representations shared between imagery and perception in the alpha frequency band we correlated (Spearman’s R) each model RDM with each participant’s neural RDM.

Additionally, to establish how well the visual and auditory models explained the organization of visual representations (within the perception task) and auditory representations (within the imagery task) respectively, we compared these models with neural RDMs extracted from classification analyses within the perception and imagery tasks (Figures S2D and S2E). For this we averaged the RDMs at time points that fell in the within task classification clusters into a single neural RDM for each task and proceeded with representational similarity analysis as described above for the cross-classification analysis.

#### Classification from broadband responses

In addition to classifying objects from oscillatory responses, we also performed conventional classification analyses [6, 86] on broadband responses (i.e., single trial raw unfiltered waveforms). These analyses followed the same logic as the classification analysis on time-frequency data, including the averaging of individual trials into pseudo-trials prior to classification analyses. As the only difference, classifiers were now solely trained and tested on response patterns across all electrodes for every time point (with the original acquisition resolution of 1000 Hz), without any frequency decomposition. As for the classification analysis on oscillatory responses, we performed a time-generalization analysis, where we cross-classified objects between perception and imagery (Figure S1G), and a within-task classification analysis, where we classified objects in each of the two tasks separately (Figure S1N).

### Quantification and Statistical Analysis

All statistical analyses were performed in MATLAB. We report results of specific tests with summary statistics and information on the test used in the Results.

The statistic of interest (mean classification accuracy or correlation coefficient in representational similarity analysis across participants, N = 38) was tested against chance level using sign permutation tests that do not make assumptions about the distribution of the data. The null hypothesis was that the statistic of interest was equal to chance (i.e., 50% classification accuracy, a Spearman’s R of 0). Under the null hypothesis, we could permute the condition labels of the EEG data, which effectively corresponds to a sign permutation test that randomly multiplies participant-specific data with +1 or −1. For each permutation sample, we recomputed the statistic of interest. Repeating this permutation procedure 10,000 times, we obtained an empirical distribution of the data, which allowed us to convert the original statistic (i.e., correlation coefficient, the time course of object classification and the time-time matrix of object classification) into p values (correlation coefficients), 1-dimensional (time courses) or 2-dimensional (time-time matrices) p value maps. We also converted the recomputed statistics to p values or p value maps (relying on the same empirical distribution as the original statistic). For the classification-based analyses we controlled the familywise error across time points using cluster size inference. All p value maps were first thresholded at p < 0.05 (cluster-definition threshold) to define supra-threshold clusters by their temporal contiguity. These supra-threshold clusters were used to construct an empirical distribution of maximum cluster size and to estimate a threshold at 5% of the right tail of this distribution. That is, the supra-threshold clusters of the original statistic were reported as significant if their size exceeded a p < 0.05. Moreover, for the classification on time-frequency resolved signals, the cluster threshold was Bonferroni-corrected for the number of frequency bands analyzed. For the correlation-based analyses we corrected p values for multiple comparisons by FDR-correction.

## Supplemental Information

Document S1. Figures S1 and S2

Document S2. Article plus Supplemental Information
