# Visual Imagery and Perception Share Neural Representations in the Alpha Frequency Band

**DOI:** 10.1016/j.cub.2020.07.023

**Published:** 2020-08-03

**Authors:** Siying Xie, Daniel Kaiser, Radoslaw M. Cichy

(Current Biology *30*, 2621–2627.e1–e5; July 6, 2020)

In the preparation of the manuscript, some citations in the article were wrongly set and thus referred to the wrong entries in the reference section. To match the citation numbers in the text with the right reference links, we rearranged parts of the references and changed the wrong numbering of citations in the Key Resources Table. The corrections are as follows:

(1) Rearranging the references of the original article: references [22–33] are rearranged as [34–45]; reference [34] is rearranged as [26]; reference [35] is rearranged as [46]; reference [36] is rearranged as [22]; references [37–39] are rearranged as [23–25]; references [40–46] are rearranged as [27–33]; references [51–53] are rearranged as [56–58]; and references [54–58] are rearranged as [51–55];
(2) Correcting citations in the Key Resources Table: citation [52] changes to [57]; citation [53] changes to [58].

These errors have now been corrected online. The authors apologize for the errors.

