# Supplementary material for: Visual Imagery and Perception Share Neural Representations in the Alpha Frequency Band
Source: Curr Biol. 2020 Jul 6;30(13):2621–2627.e5. doi: 10.1016/j.cub.2020.04.074 (PMC7342016; doi:10.1016/j.cub.2020.04.074)
Supplement: Document S1. Figures S1 and S2 [file mmc1.pdf]

Current Biology, Volume *30*

Supplemental Information

Visual Imagery and Perception Share Neural Representations in the Alpha Frequency Band

Siying Xie, Daniel Kaiser, and Radoslaw M. Cichy

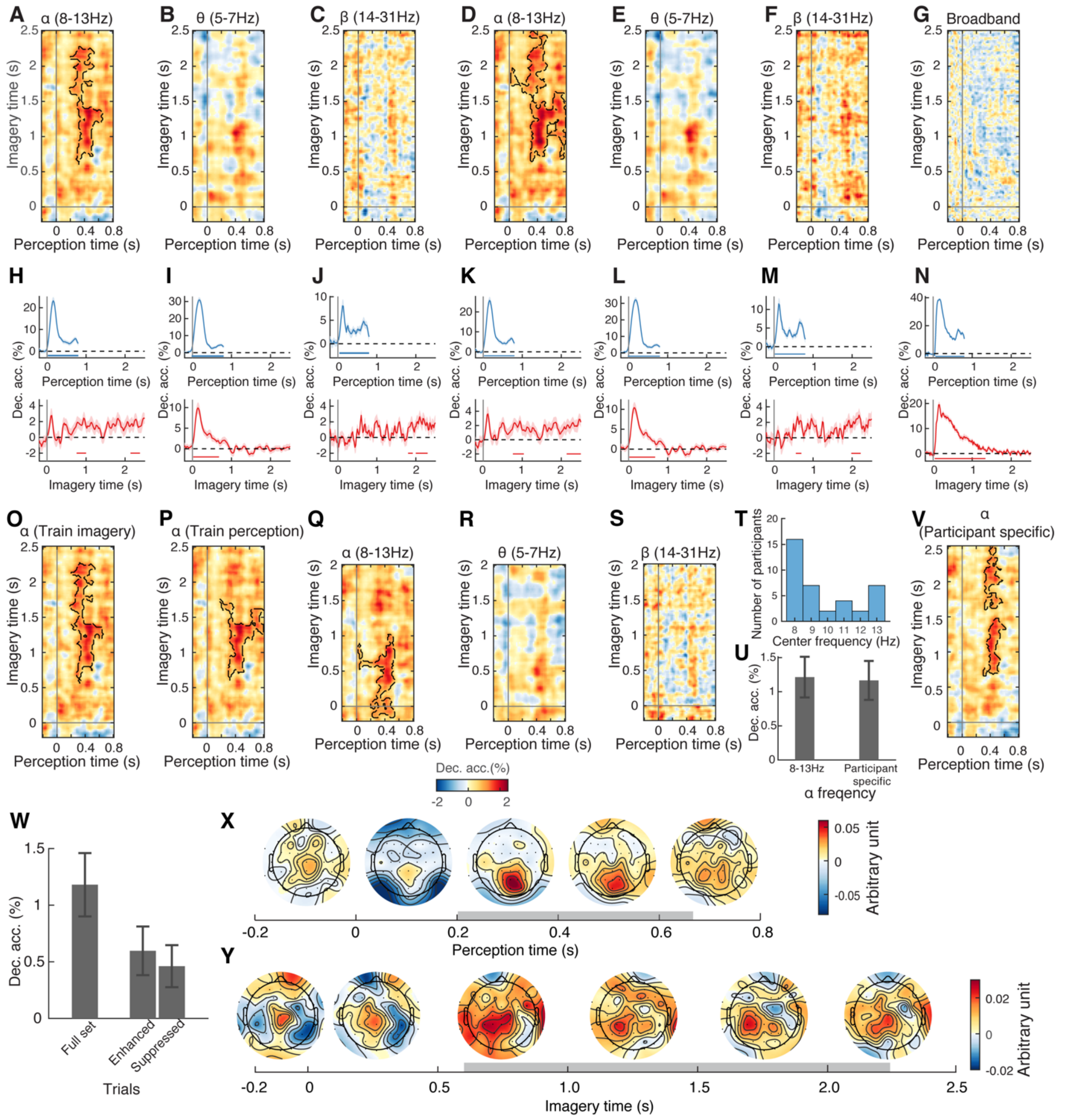

Figure S1. Analysis of object representations shared between imagery and perception, Related to Figure 1. A-C) Object cross-classification results for the alpha (A) (same as Figure 1F), theta (B), and beta (C) frequency bands. Shared representations between imagery and perception were only found in the alpha frequency band. Black outlines indicate time-point combinations with above-chance classification ($N = 38$, non-parametric sign permutation tests, cluster-definition threshold $P < 0.05$, cluster threshold $P < 0.05$ Bonferroni-corrected by 3 for the number of frequency bands tested). Dec. acc. = Decoding accuracy. **D-F)** Same as A-C) but with an alternative data aggregation procedure. Instead of averaging data across frequencies in every frequency band, data for all frequencies were entered as separate features into the classifier. Again, shared representations were only found in the alpha frequency band. **G)** Cross-classification based on broadband (evoked) responses did not reveal any shared representations. **H-N)**

Object classification results separately for the perception task (top row) and the imagery task (bottom row), corresponding to each of the analyses in A-G). For these analyses, training and testing time were yoked, yielding a single time series of classification for each task. Significant classification in the perception task was found across all frequency bands, as well as in the broadband responses. Similarly, classification was significant for all analyses in the imagery task, with a more temporally sustained classification across the epoch emerging in the alpha frequency band. Error margins denote standard errors of the mean. **O-P)** Object cross-classification results for both train-test directions, with training on either the imagery task (O) or the perception task (P). Results were qualitatively similar for both train-test directions. **Q-S)** Object cross-classification for the alpha (Q), theta (R), and beta (S) frequency bands when the imagery task data were time-locked to the offset, rather than the onset of the auditory word stimulus. Results were qualitatively similar to onset-locked analysis, revealing shared representations only in the alpha frequency band. **T-V)** Cross-decoding analysis based on subject-specific rather than canonical alpha frequencies bands. For each participant, that participant's peak alpha frequency was selected from the perception task as the frequency (with 1 Hz resolution) that allowed for the best object classification within the perception task. The subject-specific alpha band was then defined as the peak frequency plus the two neighboring frequencies of +/- 1 Hz. A histogram of the subject-specific peak frequencies is shown in (T). Object-cross classification analysis based on the subject-specific alpha frequency bands did not yield higher classification accuracy (averaged across the time-point combinations in the significant cluster) (U), while yielding qualitatively similar results as cross-classification based on canonical alpha band definition in the full time-time analysis (V). **W)** Object cross-decoding results for alpha-enhanced and alpha-suppressed perception trials, within the temporal cluster previously identified for shared representations. Trials with enhanced alpha power (relative to baseline) and suppressed alpha power (relative to baseline) did not yield different classification accuracies, suggesting that the information shared between imagery and perception is not primarily related to net alpha power. **X-Y)** Topography of classifier weights for classifiers trained on discriminating the objects from alpha activity in the perception task (X) or the imagery task (Y), for discrete time bins of 200ms in perception and 500ms in imagery, as shown below the topographies. The distribution of weights reveals the relative importance of posterior sensors in both tasks, suggesting that the shared representations are related to parieto-occipital alpha sources. Shaded bars indicate the perception and imagery times covered by the alpha frequency band cluster previously identified for shared representations.

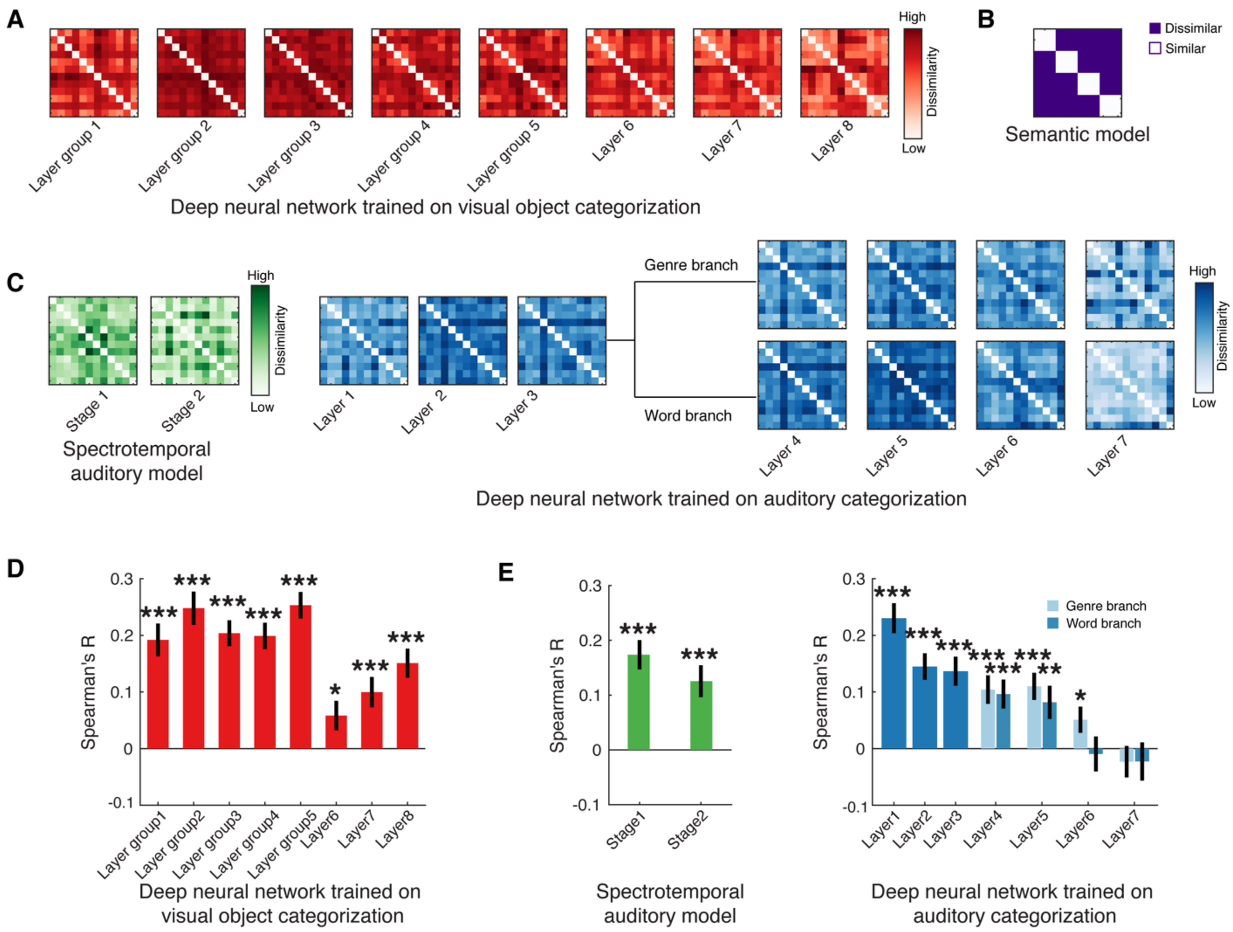

Figure S2. Analysis of the format of object representations shared between perception an imagery, Related to Figure 2. A-C) Representational dissimilarity matrices (RDMs) extracted from the three types of computational models as shown in Fig. 2. For all models, RDM entries reflect the dissimilarity between each pair of objects. **D-E)** To compare the different models to the neural representations, we correlated (Spearman's R) each model RDM with a neural RDM extracted from the alpha cluster of shared representations between imagery and perception (Figure 1F; see STAR Methods). In addition to performing this analysis for the shared representations (Figure 2B-D), we also checked how the visual and auditory models explained representations in the perception and imagery tasks, respectively. As expected, the visual model explained the objects' neural representations in the perception task (D) and the auditory models explained the objects' neural representations in the imagery task (E). Error bars reflect standard errors of the mean. Asterisks indicate significant correlations between model RDMs and neural RDMs (N = 38, non-parametric sign-permutation tests, *: $P < 0.05$, **: $P < 0.01$, ***: $P < 0.001$; FDR-corrected for multiple comparisons across RDMs per model).
